# Supplementary material for: Nutritional Care for Institutionalized Persons with Dementia: An Integrative Review
Source: Int J Environ Res Public Health. 2023 Sep 15;20(18):6763. doi: 10.3390/ijerph20186763 (PMC10531301; doi:10.3390/ijerph20186763)
Supplement: Supplementary file 1 [file ijerph-20-06763-s001.zip › ijerph-2370728-supplementary.pdf]

TABLE S1. INFORMATION ABOUT THE INCLUDED STUDIES

AUTHOR	YEAR	TITLE	STUDY PURPOSE	SAMPLE SIZE	QUALITY			
					MMAT	PRISMA	CASP	JBI
Abdelhamid, et al. [21]	2016	Effectiveness of interventions to directly support food and drink intake in people with dementia: systematic review and meta-analysis	To evaluate the available evidence on the effectiveness of interventions to improve, maintain or facilitate oral food and beverage intake, nutrition and hydration status, in people with cognitive impairment in different settings and with different levels of care and support	43 controlled interventions		27/27		
Anantapong, et al. [22]	2020	Mapping and understanding the decision-making process for providing nutrition and hydration to people living with dementia: a systematic review	To search the available evidence on the decision-making procedures for nutrition and hydration of people with dementia from the perceptions of the included participants	45 studies		27/27		
Arahata, et al. [23]	2017	A comprehensive intervention following the clinical pathway of eating and swallowing disorder in the elderly with dementia: historically controlled study	To develop and evaluate a medical care system based on the Comprehensive Geriatric Assessment for improving clinical outcomes in patients with dementia with severe eating and nutritional problems	102 elderly patients with dementia with eating and swallowing disorders.	3: 5/5			
Batchelor, et al. [24]	2017	Experimental Comparison of Efficacy for Three Handfeeding Techniques in Dementia	To establish the comparison of the efficacy levels of three different handfeeding techniques for assisting nursing home people with dementia with their meals	30 nursing home residents with advanced dementia.	2: 5/5			
Batchelor-M. et al. [25]	2019	Impact of Cognition and Handfeeding Assistance on Nutritional Intake for Nursing Home Residents.	To examine the effect of resident cognitive status and level of feeding aid provided by the nursing home personnel on resident's daily dietary intake and body weight	942 residents of 27 nursing homes	5:5/5			
Benigas, et. al. [26]	2016	Using Spaced Retrieval With External Aids to Improve Use of Compensatory Strategies During Eating for Persons With Dementia	To establish whether spaced retrieval, combined with an outside memory aid, is an effective technique for instructing persons with dementia to use compensatory swallowing techniques	5 participants with dementia	4:5/5			
Bunn, et. al. [27]	2016	Effectiveness of interventions to indirectly support food and drink intake in people with dementia:	To assess the available evidence on the effectiveness of interventions aimed at	56 interventions reported in 51 studies.		25/27		

		Eating and Drinking Well IN dementia (EDWINA) systematic review	improving food and beverage intake, through modification of the meal service or dining room context, education, exercise, or behavioral approaches in people with cognitive impairment or different types of dementia living in different settings and having different levels of care and support					
Chang, et al. [10]	2017	Prevalence and factors associated with food intake difficulties among residents with dementia	To validate the Chinese Eating Difficulty Index for the assessment of the frequency of food intake difficulties and the associated factors to eating performance in people with dementia who live in long term care residences	213 residents of long-term care facilities who has dementia.	3: 5/5			
Chen, et al. [9]	2016	Effects of a feeding intervention in patients with Alzheimer's disease and dysphagia	To analyze the effects of a dietary intervention in patients with Alzheimer's disease who suffer from dysphagia	30 nursing home residents with Alzheimer's disease with dysphagia	3:5/5			
Cipriani, et al. [6]	2016	Eating Behaviors and Dietary Changes in Patients With Dementia	To search the evidence on generalized difficulties with nutrition, diet, feeding, and eating recounted among patients with dementia	89 articles				11/11
Faraday, et al. [11]	2019	Equipping nurses and care staff to manage mealtime difficulties in people with dementia: A systematic scoping review of training needs and interventions	To examine the extent to which reported interventions for the management of eating difficulties of people with dementia meet the needs of nurses and care staff for managing the feeding difficulties of people with dementia	76 articles		21/22		
Featherstone, et al. [12]	2019	Routines of resistance: an ethnography of the care of people living with dementia in acute hospital wards and its consequences	To explore the ways in which resistance to patients with dementia care is manifested in hospital acute care setting wards and how it is classified and managed by healthcare staff	20 months observations and interviews in acute hospital wards of 5 national health service hospitals	1 :5/5			
Herke, M. [28]	2018	Environmental and behavioral modifications for improving food and fluid intake in people with dementia	To evaluate the evidence on the effects of environmental or behavioral changes on food and fluid intake, and nutritional status in people with dementia	15 studies		27/27		
Johansson, et.al. [29]	2017	Staff views on how to improve mealtimes for elderly people with dementia living at home	To describe staff opinions on how to enhance mealtimes for	22 staff members in 4 focus groups	1:4/5			

			people with dementia who continue living in their homes					
Jung et al. [30]	2020	Feasibility of a Mobile Meal Assistance Program for Direct Care Workers in Long-Term Care Facilities in South Korea	To estimate how valid and preliminarily effective is an application of a meal attendant training program for its use by healthcare personnel caring for people with dementia in long-term care facilities in South Korea is	23 dyads of people with dementia caregivers in a long-term care facility. The focus group included 6 healthcare staff members and 5 nurses	5:5/5			
Kawaharada et al. [31]	2019	Impact of loss of independence in basic activities of daily living on caregiver burden in patients with Alzheimer's disease: A retrospective cohort study	To establish the association between impaired performance in basic activities of daily living of people at different stages of Alzheimer's disease with the perceived burden of their caregivers	117 outpatients with Alzheimer's disease and their caregivers	3:5/5			
Keller et al. [13]	2018	Development and Inter-Rater Reliability of the Mealtime Scan for Long-Term Care	To create and validate the reliability the Mealtime Scan for assessing mealtime experience in long term care facilities.	10 dining rooms in 3 long-term facilities	4: 5/5			
Keller, H.[32]	2016	Improving food intake in persons living with dementia	To review the literature on nutritional status in persons diagnosed with dementia	74 studies				4/11
Kobayashi,et al. [33]	2017	Prevalence of oral health-related conditions that could trigger accidents for patients with moderate-to-severe dementia	To establish the prevalence of oral health situation undetected by health care professionals that raise risks of negative events in hospitalized patients with different levels of dementia	92 hospitalized dementia patients in the dysphagia rehabilitation service	4:5/5			
Leah,V. [34]	2016	Supporting people with dementia to eat	To search for evidence on the best ways of helping people with dementia to eat	22 studies		25/27		
Li, et al. [35]	2020	Overview of systematic reviews: Effectiveness of non-pharmacological interventions for eating difficulties in people with dementia	To search the available evidence for assessing the effectiveness of non-pharmacological strategies for dealing with eating problems in people with dementia	18 systematic reviews		23/27		
Li, et al.[36]	2020	Informal dementia caregivers' experiences and perceptions about mealtime care: A qualitative evidence synthesis	To analyze qualitative evidence on the mealtime care experiences of non-formal caregivers of people with dementia	10 qualitative studies			10/10	
Liu, et al. [4]	2016	Factors associated with eating performance for long-term care residents with moderate-to-severe cognitive impairment	To analyze the relationship of specific personal and context factors with eating performance in long term care residents with different types of cognitive impairment	199 residents in 8 long term care facilities	3:5/5			

Liu, et al. [37]	2017	The association of eating performance and environmental stimulation among older adults with dementia in nursing homes: A secondary analysis	To analyze the relationship of the context stimulation with the eating performance in long term care residents who had dementia	Analysis of 36 videos of the staff members -residents with dementia interactions	3:5/5			
Liu, et al. [38]	2019	Factors influencing the pace of food intake for nursing home residents with dementia: Resident characteristics, staff mealtime assistance and environmental stimulation	To analyze the relationship of the rate of food intake in residents with dementia with their characteristics, mealtime care and the contextual stimulation they receive	Analysis of 36 videos of 19 staff members and 15 residents with dementia in 8 long term care facilities	3:5/5			
Liu, et al. [39]	2020	Association between Intake of Energy and Macronutrients and Memory Impairment Severity in US Older Adults, National Health and Nutrition Examination Survey 2011-2014	To analyze the relationship between intake of energy and macronutrients with memory damage among older adults in the united States	Data form the National Health and Nutrition Examination Survey that included 3623 older adults	3:5/5			
Liu, et al.[40]	2020	Facilitators and barriers to optimizing eating performance among cognitively impaired older adults: A qualitative study of nursing assistants' perspectives	To examine the perceptions of nursing assistance on the different facilitators and barriers that exist to improve mealtime performance of people with cognitive impairment	6 focus groups with 23 nursing assistants in 2 nursing homes and 1 hospital.	4:5/5			
Liu, et al.[41]	2020	Ease of use, feasibility and inter-rater reliability of the refined Cue Utilization and Engagement in Dementia (CUED) mealtime video-coding scheme	To refine the Utilization and Engagement in Dementia mealtime video coding scheme for the assessment of the mealtime intake and residents with dementia – health care staff interactions	Analysis of 110 videos with 25 residents with dementia and 29 staff members	4:5/5			
Liu, et al. [42]	2021	Nutrition and exercise interventions could ameliorate age-related cognitive decline: a meta-analysis of randomized controlled trials.	To assess the available evidence on nutritional interventions linked with physical exercise effect on age-related cognitive decline	6 randomized controlled trials that included 1039 participants.		26/27		
Marples et al. [43]	2017	The effect of nutrition training for health care staff on learner and patient outcomes in adults: a systematic review and meta-analysis	To assess the literature on influence of different nutritional health care training strategies on patient outcomes and caring knowledge, attitude, and practice	24 studies		27/27		
Martin et al. [8]	2018	Body composition, dietary, and gustatory function assessment in people with Alzheimer's disease	To analyze the relationship among food, diet and exercise habits, body composition, and gustatory function in people with Alzheimer´s disease	75 patients with Alzheimer´s disease	3:5/5			
McGrattan et al [44]	2021	A mixed methods pilot randomized controlled trial to develop and evaluate the feasibility of a	To estimate the viability of the THINK-MED intervention that includes Mediterranean diet and	10 participants	2:5/5			

		Mediterranean diet and lifestyle education intervention 'THINK-MED' among people with cognitive impairment	lifestyle education for older adults with cognitive impairment					
Murphy et al. [45]	2017	Nutrition and dementia care: developing an evidence-based model for nutritional care in nursing homes	To develop an evidence-based model for guiding nutritional care of people with dementia living in a nursing home	9 focus groups and 5 semi-structured interviews with health care nursing home staff and family caregivers	1:5/5			
Nell et al. [5]	2016	Factors affecting optimal nutrition and hydration for people living in specialized dementia care units: A qualitative study of staff caregivers' perception	To search the perceptions of health care staff members on factors influencing nutrition and hydration of residents of specialized dementia care units in New Zealand	11 staff caregivers at 2 specialized dementia care Units	1:5/5			
Palese et al. [46]	2018	Interventions maintaining eating Independence in nursing home residents: a multicenter qualitative study	To search for interventions offered by healthcare staff to support nursing home residents nutrition, that have not been documented	13 focus groups with 54 healthcare professionals	1:5/5			
Palese et al. [47]	2020	Enhancing independent eating among older adults with dementia: a scoping review of the state of the conceptual and research literature	To describe the available evidence for maintaining and promoting autonomy while eating in nursing homes residents with dementia	17 reviews			17/18	
Painter, et al. [48]	2017	Texture-modified food and fluids in dementia and residential aged care facilities	To evaluate the evidence for texture-modified food and fluids for long term care older residents with dementia	22 studies		23/27		
Park, et al. [49]	2018	National study of the nutritional status of Korean older adults with dementia who are living in long-term care settings	To assess the nutritional status of long-term care older residents with dementia in Korea	Data from the Nationwide Survey on Dementia Care in Korea that included 3472 older adults with dementia	3:5/5			
Poscia, et al. [50]	2018	Effectiveness of nutritional interventions addressed to elderly persons: umbrella systematic review with meta-analysis	To review the available evidence on nutritional interventions to promote healthy aging in older individuals	28 papers in 6 databases		23/27		
Prizer,et al. [51]	2018	Progressive Support for Activities of Daily Living for Persons Living With Dementia	To review the available evidence on practices for caring for the basic daily needs of people with different levels of dementia	59 documents				8/11
Quinn et al. [52]	2019	Influence of Positive Aspects of Dementia Caregiving on Caregivers' Well-Being: A Systematic Review	To search the literature on how positive aspects of caregiving impacts well-being of the caregivers of people who has dementia	53 studies		23/27		
Saarela et al. [53]	2017	Changes in malnutrition and quality of nutritional care among aged residents in all nursing homes and	To describe and compare the nutritional status and the nutritional care quality of	Information from 4 databases of the residents in nursing homes and assisted living facilities from	4:5/5			

		assisted living facilities in Helsinki 2003–2011	Helsinki residents of nursing homes and assisted living facilities 2003 – 2011	2003 -2011, in Helsinki, Finland. They include 1987, 1377, 1576 and 1585 subjects, respectively				
Salminen et al. [54]	2019	Energy Intake and Severity of Dementia Are Both Associated with Health-Related Quality of Life among Older Long-Term Care Residents	To explore the association between modification of energy intake and health-related quality of life in institutionalized older people with different stages of dementia	538 older residents with dementia.	3:5/5			
Shatenstein et al. [55]	2017	Outcome of a Targeted Nutritional Intervention Among Older Adults With Early-Stage Alzheimer's Disease: The Nutrition Intervention Study	To evaluate the effect of a targeted nutritional intervention directed to older adults with early-stage Alzheimer's disease in a community setting on their nutritional intake, weight, and strength	67 dyads (older adults with Alzheimer's disease and their caregivers).	3:5/5			
Sheppard,et AL.[56]	2016	A Systematic Review of Montessori-Based Activities for Persons With Dementia	To assess the best available evidence guided by the Montessori conceptual framework activities for people with dementia	14 articles		20/27		
Simmons,et al. [57]	2018	A Quality Improvement System to Manage Feeding Assistance Care in Assisted-Living	To describe a quality improvement system for people with dementia nutritional care in an assisted- living facility	53 residents	4: 4/5			
Snyder,et al. [58]	2020	Caregiver Psychological Distress: Longitudinal Relationships With Physical Activity and Diet	To compare the psychological distress and health behaviors of a group of caregivers of spouses with Alzheimer's disease with a group of non-caregivers, over 2 years	122 caregivers of spouses with Alzheimer disease, and 117 non-caregivers	3:5/5			
Soininen,et al. [59]	2017	24-month intervention with a specific multinutrient in people with prodromal Alzheimer's disease (LipiDiDiet):a randomized, double-blind, controlled trial	To report a 24-month follow-up of an intervention with LipiDiDiet in people with prodromal Alzheimer's disease	296 participants complete the trial	2:5/5			
Soininen,et al. [60]	2021	36-month LipiDiDiet multinutrient clinical trial in prodromal Alzheimer's disease	To report a 36-month follow-up of a intervention with LipiDiDiet in people with prodromal Alzheimer's disease	162 participants complete the trial	2:5/5			
Takada, et al. [61]	2017	Grouped factors of the 'SSADE: signs and symptoms accompanying dementia while eating 'and nutritional status—An analysis of older people receiving nutritional care in long-term care facilities in Japan	To validate the SAADE assessment tool in the detection of mealtime signs and symptoms associated with dementia and the nutritional status of older adults receiving care in long-term institutions in Japan	259 older residents in 14 long-term care facilities	3:5/5			

Tangvik, et al. [62]	2021	Effects of oral nutrition supplements in persons with dementia: A systematic review	To evaluate the available evidence on the effects of oral nutrition supplements on nutritional intake and status, and cognitive and physical outcomes of older people with dementia	10 clinic essays		27/27		
Tombini, et al. [63]	2016	Nutritional Status of Patients with Alzheimer's Disease and Their Caregivers	To analyze the nutritional status of the dyads of patients with Alzheimer's disease living at home and their caregivers and the influence of different factors on their nutrition	90 dyads (patients with Alzheimer's disease - caregivers)	4:5/5			
Watanabe, et al. [64]	2019	Association between dysphagia risk and unplanned hospitalization in older patients receiving home medical care	To associate the dysphagia risk and unplanned first hospitalization in older people with cognitive alterations receiving home medical care during	Data from Nagoya Elderly with Home Medical study that included 178 patients	4:5/5			
Whitelock, et al. [65]	2018	On your own: older adults' food choice and dietary habits	To describe older adults' perceptions and habits related to dietary activities and factors influencing their food choice	30 participants from 63 – 90 years	1:5/5			
Wu, et al. [66]	2018	Mixed methods developmental evaluation of the CHOICE program: a relationship-centered mealtime intervention for long-term care	To evaluate the CHOICE program design and implementation for the improvement of the mealtime experience in long term care facility	64 residents and 25 long term care staff	5:5/5			

Source: Authors elaboration based on the studies that were included in the review, 2023.
